# Comprehensive approaches to addressing mental health needs and enhancing school security: a hybrid type II cluster randomized trial

**DOI:** 10.1186/s40352-020-0104-y

**Published:** 2020-01-14

**Authors:** Andria B. Eisman, Justin Heinze, Amy M. Kilbourne, Susan Franzen, Christopher Melde, Edmund McGarrell

**Affiliations:** 1 Department of Health Behavior and Health Education, School of Public Health, University of Michigan, 1415 Washington Heights, Ann Arbor, MI 48109 USA; 2 Department of Psychiatry, University of Michigan Medical School, Ann Arbor, MI USA; 3 Quality Enhancement Research Initiative, U.S. Department of Veterans Affairs Center for Clinical Management Research, North Campus Research Complex (NCRC), 2800 Plymouth Road, Building 16, Ann Arbor, MI 48109-2800 USA; 4 Michigan State Unversity, School of Criminal Justice, Baker Hall, 655 Auditorium Road, Room 557, East Lansing, MI 48824 USA

**Keywords:** School, health, Restorative justice, Implementation, Intervention study, Violence, Mental health, Children

## Abstract

**Background:**

School safety is fundamental to fostering positive outcomes for children. Violence remains a critical public health issue with 8.1% of elementary and 21.8% of middle school students reporting daily or weekly bullying in 2015–16. Similarly, over half of lifetime mental health concerns become evident before age 14. Thus, elementary school is a key time for comprehensive school safety interventions. Yet, interventions are rarely delivered with fidelity in community settings. Evidence-based interventions must be complemented by implementation strategies to achieve desired public health outcomes.

**Methods:**

We develop and test an intervention focused on promoting a positive school climate guided by a school-based 3-person leadership team (3-PLT) using a hybrid Type II design. The 3-PLT includes a School Resource Officer, (SRO), administrator and mental health services professional as a newly appointed climate specialist (CS). The interventions to be delivered include 1) Restorative justice, 2) Mental Health First Aid and 3) Crime Prevention Through Environmental Design. The CS will lead the team and coordinate implementation through a process of interactive problem solving and supports, consistent with the implementation facilitation strategy. We will conduct a cluster randomized controlled trial with staged entry over two school years in Genesee County, Michigan (*n* = 20 elementary schools, with 10 participating per school year). We will use a combination of data sources including data collected by schools (e.g., discipline data), a student survey, and a teacher survey. We will also conduct a process evaluation and assess implementation and sustainability through focus groups with key stakeholders, teachers, and students. Finally, we will conduct a cost-benefit analysis.

**Discussion:**

Results from both the behavioral outcome and implementation strategy evaluations are expected to have significant implications for school safety and student well-being. This study adopts a unique approach by integrating three evidence-based programs and incorporating implementation facilitation led by the CS as part of the 3-PLT to support intervention delivery and enhance public health impact among schools in disadvantaged communities with students at risk of poor health outcomes. This study aims to create a comprehensive, well-integrated model intervention that is sustainable and can be translated to similar high-risk settings.

**Trial registration:**

Trial was retrospectively registered, registration ISRCTN1226421, May 16, 2019.

## Background

School safety is fundamental to fostering positive short and long-term outcomes for children, including positive mental health, school connectedness, student retention and academic success (Battistich, Schaps, & Wilson, 2003; Horner et al., 2009). We define a safe school as one that minimizes violence, promotes student mental health, and fosters a social climate that promotes positive development. Unfortunately, concerning rates of school violence persist in the US: in 2017, 19.0% of students were bullied, 15.7% carried a weapon at least once in a month (3.8% on school property), and 6% had been threatened or injured with a weapon (Kann et al., 2018). Rates of violent, aggressive, and bullying behaviors are similarly concerning among younger students with 8.1% of elementary and 21.8% of middle school students reporting daily or weekly bullying in 2015–16 (Diliberti, Jackson, & Kemp, 2017). Violence victimization is associated with distress, adjustment difficulties, and mental health problems. Exposure to violence, including direct victimization and well as exposure in the school environment, is a potent risk factor for poor mental health outcomes including depression and anxiety (Kennedy, Bybee, Sullivan, & Greeson, 2010).

The CDC (Perou et al., 2013) identifies mental health problems, including depression and anxiety, as a critical public health issue among youth with significant impact on the individual, family, and community. Mental health issues that go untreated early in life are associated with further problems, including increased likelihood of academic failure, dropout, substance use, relationship conflicts, violence, and suicide (World Health Organization, 2012). In the short term, mental health problems evident in middle school predicts school absences a year later (Suldo, Thalji, & Ferron, 2011). In addition, many children, particularly those living in low resource communities, experience disproportionate risk of violence and subsequent mental health consequences without sufficient treatment and prevention services needed to reduce risk of poor outcomes (O’Connell, Boat, & Warner, 2009). Childhood vulnerability is exacerbated in high stress environments when children and youth receive limited support from adults (O’Connell et al., 2009). Yearly, an estimated 13–20% of children aged 3–17 years experience a mental disorder, and more than half of lifetime psychiatric diagnoses have an initial age of onset before age 14 (Kessler et al., 2005).

School climate plays a major role in shaping the lives of students, affecting violence (Brookmeyer, Fanti, & Henrich, 2006), mental health and wellness (Jacobson & Rowe, 1999), truancy and achievement (Astor, Guerra, & Van Acker, 2010). The National School Climate Council recommends an encompassing definition of school climate that includes experiences of school life, that reflect the norms, goals, values, teaching, organization structure, and relationships. Relationships include connections among students, teachers, and staff; feelings of commitment to the institution; and connection to a community (Pittman & Richmond, 2007).

The relationship between student outcomes and school climate are evident longitudinally (Anderman, 2002; Goodman, 1997). Researchers have found that poor school-based relationships were associated with initiation of deviant behavior (Dornbusch, Erickson, Laird, & Wong, 2001; McNeely & Falci, 2004). Consequently, promoting a positive school climate is an important mechanism by which interventions can reduce risk of poor health outcomes including violence and mental distress. Researchers suggest that schools, particularly those with concentrated poverty, may benefit from multicomponent prevention approaches that improve positive discipline management and support positive psychosocial climates, effectively identify youth experiencing mental distress and improves the physical as well as social environment (Gottfredson, Gottfredson, Payne, & Gottfredson, 2005).

In order to effectively address challenging issues such as school safety, communities need to deliver multicomponent interventions targeting prevention efforts across levels of social ecology (Komro, Flay, Biglan, & Wagenaar, 2016; PriCowan, Vaillancourt, Rossen, & Pollitt, 2013). Even the best individual interventions have relatively limited scope in terms of outcomes when offered alone, and, consequently, small effects when taken to scale; therefore, multicomponent interventions have greater potential to achieve positive outcomes at the school or community level than a single intervention alone (Komro et al., 2016). Evidence-based interventions (EBIs) that address the multifaceted nature of school safety such as Restorative Justice (RJ), Mental Health First Aid (MHFA) and Crime Prevention Through Environmental Design (CPTED) when deloyed as a single, coordinated, multicomponent intervention approach, are promising approaches to improving the school environment.

Yet, more complex, multicomponent interventions are also more challenging to implement. Such interventions require effective implementation strategies to adopt the constellation of EBIs and adapt them to suit the needs of the context, providers and target population. Interventions will fail to achieve their desired effects if not implemented well (Durlak, 2015). Researchers have acknowledged that evidence-based interventions must be complemented by implementation strategies to achieve desired public health outcomes (Kirchner, Waltz, Powell, Smith, & Proctor, 2018). Implementation strategies are highly specified, theory-based methods to enhance EBI delivery in community settings and are key to bridging the research-to-practice gap (Kilbourne et al., 2014). Study designs that assess implementation strategy utility *and* evaluate EBI effectiveness, such as with hybrid designs, provide vital information for stakeholders about using implementation strategies with new innovations to maximize public health impact.

**Fig. 1:**
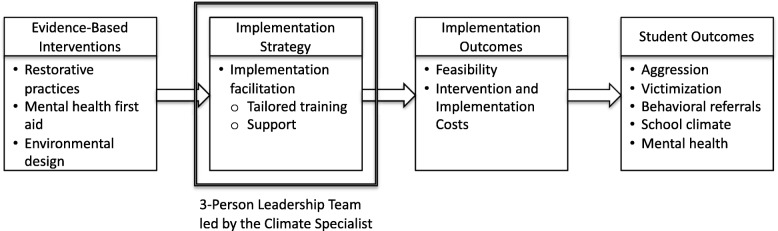
Proposed Conceptual Relationship Between Evidence-Based Interventions, Implementation Strategies and Study Outcomes. Adapted from Proctor et al. (2009) and Lyon (Lyon, 2018)

To inform the optimal implementation strategies for these effective interventions, we develop and test an intervention focused on promoting a positive school climate guided by a school-based 3-person leadership team (3-PLT) using a hybrid Type II design. A hybrid Type II design tests the effectiveness of the intervention and determines the feasibility and potential utility of an implementation strategy (Curran, Bauer, Mittman, Pyne, & Stetler, 2012). The 3-PLT includes representatives from the police (School Resource Officer, SRO), school (e.g., administration), and mental health services (i.e., social work), the latter of whom leads the team as a newly appointed climate specialist (CS). The team, led by the CS, work together to support the integration of the key intervention components: (1) RJ practices, (2) MHFA training, and (3) CPTED (see Fig. 1). The CS coordinates these efforts as a staff member within the school through a process of interactive problem solving and supports, consistent with the implementation facilitation strategy (Ritchie, Dollar, Kearney, & Kirchner, 2014). We will focus on change among students in an early developmental period—elementary school students aged 8–12 years—in a county with significant social and economic challenges. The purpose of this research is to study the effectiveness and implementation of three complementary interventions delivered concurrently to enhance school safety through improved school climate. School climate represents a critical mechanism by which interventions, including multicomponent school safety and mental health interventions, can reduce risk of violence and mental distress among youth.

## Methods/design

### Aims and objectives

The overarching objective of this study is to provide a safe school environment to positively impact health, wellbeing, social, educational, violence and delinquency outcomes among youth. This is accomplished through the concurrent delivery of a multicomponent approach consisting of 3 integrated interventions: Restorative Justice (RJ), Mental Health First Aid (MHFA) and Crime Prevention Through Environmental Design (CPTED). The implementation strategy used is a facilitation approach based on the Implementation Facilitation, Enhanced REP, and the iPARiHS framework (Kilbourne et al., 2013; Ritchie et al., 2014) as part of the 3-PLT (see Fig. 1). Elementary school safety is understudied yet represents a critical period in which to develop positive mental health, build constructive and trusting relationships with adults, and prevent early experiences of violence.

### Primary study aim

The primary study aim is to examine the overall effectiveness of the intervention, including change in violence (e.g., fights, bullying, victimization), over time compared with a control group of students who receive school practice as usual.

### Secondary study aim 1

Employ implementation facilitation from an appointed school climate specialist (CS) to support delivery of a multicomponent, integrated intervention and evaluate feasibility and potential utility to support sustainment.

### Secondary study aim 2

Examine specific mechanisms associated with change in mental health (e.g., anxiety, depression, well-being), including perceptions of school climate as a moderator.

### Secondary study aim 3

Estimate the costs of the intervention and its implementation and conduct a cost-benefit analysis for positive outcomes, such as improve school climate perceptions, associated with participation in the interventions.

### Methods

**Fig. 2:**
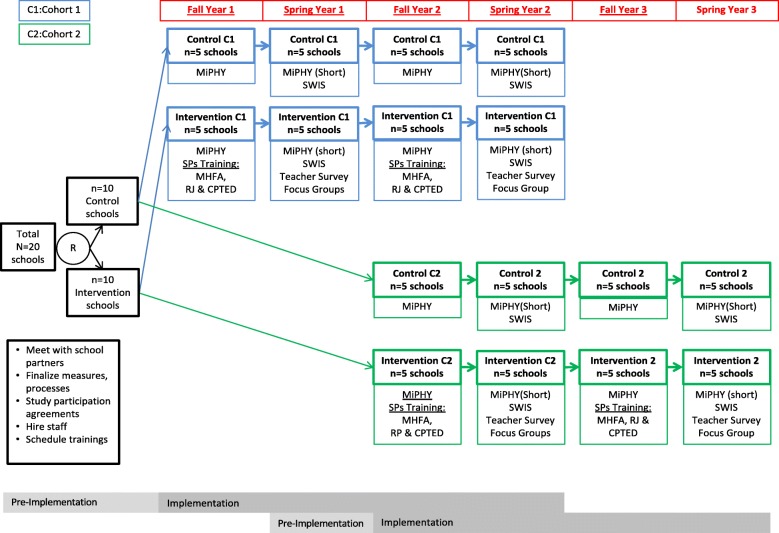
Cluster RCT of the Comprehensive School Safety Intervention delivered using the implementation facilitation strategy

The cluster randomized trial evaluates a school safety intervention in a community with significant need, Genesee County, Michigan (see Fig. 2). This study was reviewed and approved by the Michigan State University Institutional Review Board (IRB# × 15-1129e). This study takes advantage of ongoing partnerships with the Genesee Intermediate School District in Michigan. We do not intend to collect data from participants who discontinue or deviate from protocols. Among the primary data sources to be collected (e.g., focus groups, interviews, teacher surveys), the data will be kept on a password protected server and de-identified. The study does not employ a data monitoring committee, but this study is structured such that the intervention team (i.e., employees of the GISD), is separate from the training team (i.e., employees of international and regional organizations, local universities, and the district wide [GISD] training team offering training in facets of the intervention), which is separate from the data collection team (i.e., employees of the two universities charged with the process and outcome evaluation, led by a co-PI), which is separate from the data analysis team (i.e., employees of the two universities charged with the process and outcome evaluation, led by a separate co-PI), providing sufficient independence and protecting against potential conflicts of interest. Thus, those tasked with training and technical assistance associated with components of the intervention are not involved in the analysis of data or reporting of outcomes given the potential bias this might engender as a result of the incentives associated with reports of a positive programmatic impact, such as profits associated with increased demand for training and technical assistance in the components of the program. There is no similar financial incentive for university-based researchers charged with the analysis of data and reporting of results, as they are not employed by any of the organizations charged with program implementation (i.e., GISD) or future dissemination of intervention components (i.e., training and technical assistance providers).

### Study setting

The current study is in Genesee County, Michigan. Widespread losses in industrial jobs in many US cities have led to unemployment, population loss and changes in land use (including high rates of vacant buildings); these demographic and economic changes have contributed to neighborhood instability, disadvantage and elevated rates of violent crime (Glaeser & Gyourko, 2005; Sampson, Raudenbush, & Earls, 1997). Genesee County, especially around the city of Flint, has lost almost 90% of the automotive industry jobs that bolstered its economy in the 1960s. The economic hub of Genesee county, Flint’s current challenges include high rates of poverty (60% of children live in poverty) (U.S. Census Bureau, 2015) and an unemployment rate that is 50% higher than the state average (Bureau of Labor Statistics, 2014). Across Flint community elementary schools, 92% of students are eligible for free or reduced lunches (Michigan Department of Education, 2017). Surrounding Genesee communities face similar disadvantage with poverty (see Table 1) and violence rates exceeding state averages. This high level of community violence has significant implications for levels of trauma and corresponding mental health issues for youth residing in Flint and surrounding Genesee County.

**Table 1:** Study Sites by demographic variables

School	Free or Reduced Lunch ^a^	Grade 3–8 Students proficient in Math and English (2017–2018)^b^	Racial/ethnic minority composition^a^
1	78%	7.6%	34%
2	86%	38.6%	31%
3	55%	15.0%	29%
4	66%	26.3%	12%
5	91%	3.4%	73%
6	76%	9.9%	16%
7	84%	10.4%	24%
8	84%	2.5%	86%
9	78%	12.9%	69%
10	55%	29.4%	31%
11	97%	14.6%	100%
12	75%	32.0%	72%
13	91%	6.4%	72%
14	68%	34.1%	40%
15	84%	15.0%	74%
16	66%	29.5%	21%
17	56%	16.9%	10%
18	52%	27.1%	96%
19	95%	3.2%	9%
20	90%	11.1%	93%
State of Michigan	45.9%	30.7%	67.0%

^a^Data provided by the partner intermediate school district, internal data

^b^Parent Dashboard for School Transparency, Center for Educational Performance and Information, https://www.mischooldata.org Accessed 9/13/2019

### School safety challenges

School safety is a major concern in Flint and Genesee Co. as a whole. In a survey of Genesee Co. 4th through 6th grade students, 42% reported seeing violence in the school once per month or more, 13% did not feel safe while at school, and 3% skipped school because they were afraid of being hurt. There were also widespread mental health challenges: 44% of students worried a lot, 43% felt nervous, 30% could not stop being sad, and more than half felt they were too tired to do things (Prevention Research Center of Michigan, 2011).

### Study design

See Fig. 2 for study flow diagram. In the pre-implementation phase, the project team will meet with intermediate school partners and individual schools, finalize processes and measures, secure formal agreements of study participation with schools, hire staff for the project and schedule all trainings. The current study is a cluster randomized trial in which each school will participate in the study over two years. The interventions will be staggered (5 intervention + 5 control schools starting in Year 1; 5 additional intervention and 5 additional control schools in Year 2) in order to maximize the likelihood of intensive implementation. It focuses on three key areas: (i) at a universal prevention level, focusing on changes to the physical school environment (CPTED) and promoting consistent and fair discipline and reward practices (RJ); (ii) a more targeted approach, to help those with early signs of mental health problems (MHFA); and (iii) engaging those involved in violent or aggressive situations at school through RJ practices. As noted above, the interventions are guided by a 3-PLT who seek to utilize the interventions to promote positive school climate.

### Sample

#### Schools

We will recruit 20 schools and implement the intervention across three school years (see Table 1). Co-educational elementary schools in Genesee County Intermediate School District (GISD), Michigan will be invited to participate. Schools participating will be similar in terms of proportion of students receiving free/reduced school lunches and approximate size of the school. Prior to the intervention roll-out, all 20 schools will be randomized to intervention or control. The intervention will be implemented in stages. Cohort 1 schools will include 5 intervention and 5 control schools. Cohort 2 will include10 additional schools (5 intervention and 5 control) for a total of 10 intervention and 10 control schools; each school will implement the intervention for two years.

#### Student eligibility and recruitment

There are 21 public school districts and 13 academies (charter schools) serving over 67,000 students in the GISD. Recruitment efforts will begin with presentations to the superintendents of the schools and academies. This will be followed up by similar presentations and briefings to school building principals and/or their designees (dean of students, academic services, teachers). The briefings explain the goals of the research project, the benefits to participating schools, anticipated intervention and research activities, eligibility, and similar issues. Eligibility includes having at least two grades between grades 4–6 and having a student population with 50% or higher free or reduced school lunch eligibility, being involved in MIBLISI (Michigan’s Integrated Behavior and Learning Support Initiative) and PBIS (Positive Behavioral Interventions & Supports) and being committed to the project. Across Genesee County, there are currently an average of 180 4th–6th graders in each school resulting in a maximum sample size of 3600 students over the course of the study (using a 66% consent/retention rate).

### Evidence-based interventions to be implemented

#### Universal and targeted prevention

*Universal strategies* address an entire population regardless of risk level or current behavior. This approach aims to reach a large number of individuals at once; it develops strategies and a supportive environment that enables all children to achieve sufficient competence to thrive, and thus prevent or reduce engagement in violence (Leshner, 1997). In contrast, *targeted strategies* are designed for individuals who meet specified risk criteria, for example, showing signs of mental illness (Leshner, 1997). Simultaneous deployment, particularly one tailored to a school’s culture, is commonly considered more likely to effect change (Bonell et al., 2010; Maddox & Prinz, 2003). The proposed intervention incorporates universal and targeted approaches facilitated by a school-based 3-PLT. The team will participate in ongoing activities and engagement with school staff and students to support and reinforce the interventions, foster school-community relationships and support sustainability. These activities will be led by the CS who will apply principles of implementation facilitation to promote effective EBI delivery and tailor the interventions to the context.

The universal and targeted prevention design reflects the integrated and comprehensive intervention and suggested best practice, i.e. is comprehensive, improves access to mental health, balances physical and psychological safety, employs a positive school discipline approach, considers and responds to each schools’ culture and context, and acknowledges that change takes time (PriCowan et al., 2013). Prior school safety interventions have tended to implement these strategies in isolation rather than integrating in a coordinated manner. The proposed research will advance comprehensive school safety and using of implementation science to enhance delivery of such programs.

#### Restorative justice (RJ)

Restorative justice is a philosophy and process that defines crime as doing harm to people and relationships, rather than simply violating the law (Zehr, 2002). Thus, it requires consideration of the victim and community during the justice process. Although the definition continues to evolve, it has most commonly been described as “a process whereby all the parties with a stake in a particular offense come together to resolve collectively how to deal with the aftermath of the offense and its implications for the future” (Marshall, 1996). Restorative practices focus on repairing the harm caused, engaging victims and relevant school community members in the decision making process, holding offenders accountable, and preventing similar actions in the future. By focusing on accountability, fairness, and situational responses to unique events, restorative justice provides a useful framework for alternatives to zero tolerance or traditional disciplinary actions for school violence. Overall, researchers have found robust support for RJ practices, including enhanced school safety, reduced discipline problems and other behavioral referrals (Karp & Breslin, 2001; Mirsky, 2011; Stinchcomb, Bazemore, & Riestenberg, 2006).

Prior research suggests that RJ practices may have particular efficacy for youth in the age ranges targeted in this project. Researchers found, among students 14 years and younger, that youths participating in family group conferences were less likely to re-offend than youths participating in other court-ordered programs (McGarrell & Hipple, 2007). Further, offending youths, parents, and victims all expressed much more favorable perceptions of the fairness, respect, and value of the conference experience (McGarrell, 2001). Long-term follow-up of this research demonstrated that conferences following principles of “restorativeness” and “procedural fairness” were associated with reduced re-offending at 24 months and 10 years following the original conference (Hipple, Gruenewald, & McGarrell, 2014, 2015).

Restorative justice programs can be implemented in a variety of ways to meet the needs of individual schools, but retain a set of common principles: (1) repair harm, (2) reduce risk, and (3) empower community (O’Brien, 2007). When applied to school environments, the restorative approach comprises an overarching philosophy and processes that build community in classrooms and entire schools and include proactive processes that aim to prevent wrongdoing (Mirsky, 2011). This may be accomplished through school-wide discipline practices promoting fairness in rules and enforcement, as well as consistency of reinforcing positive behavior, and may include more targeted approaches like peer mediation (Bazemore & Umbreit, 2004; Reimer, 2011). Peer mediation involves a facilitated discussion with multiple stakeholders including the offender, victims, family members, friends, school personnel, and community members. It is typically used in response to a specific offense, to explore what happened, address and repair the harm done, and determine strategies for preventing it in the future. The 3-PLT and particularly the mental health expert will lead many of the proposed peer mediation and restorative justice components of the program and it aligns closely with the support in the MHFA and climate change associated with CPTED. Within this framework, individual schools can tailor the approach as needed for their school and more specifically for each mediation need.

#### Mental health first aid (MHFA)

MHFA is defined as help provided in the context of a mental health problem or mental health crisis; importantly, the help is provided until appropriate professional help is received or the crisis is resolved (Yap & Jorm, 2011). The Substance Abuse and Mental Health Services Administration (SAMSHA) (SAMHSA National Registry of Evidence-based Programs and Practices, n.d.) lists MHFA as part of its National Registry of Evidence-based Programs and Practices. Trainers deliver an 8-h session not intended to teach therapeutic skills but rather to raise awareness of mental illness symptoms and build skills in providing initial help and guiding someone toward treatment (Kitchener & Jorm, 2006). It includes highlighting evidence-supported treatment and engaging individual resources (e.g., family).

Qualitative studies and randomized controlled trials of MHFA indicate greater confidence to help, less stigma around mental illness, better recognition of symptoms, and improved perceived value (Jorm, Blewitt, Griffiths, Kitchener, & Parslow, 2005; Jorm, Morgan, & Wright, 2008; Kelly et al., 2011; Yap & Jorm, 2011, 2012; Yap, Wright, & Jorm, 2011). Research examining MHFA has focused on adults and adolescents, yet preadolescents are also in need of timely and appropriate mental health services, suggesting value in evaluating and validating an extension of the approach for preadolescents.

Teachers are typically not trained in mental health (Reinke, Stormont, Herman, Puri, & Goel, 2011) but are in a position to identify early symptoms, which if managed promptly can substantially reduce negative outcomes and healthcare costs (Knapp, McDaid, & Parsonage, 2011). MHFA training may support teacher needs by providing resources and skills as well as debunking misconceptions and myths which may otherwise reduce helping behavior (Yap & Jorm, 2011). Teachers typically do not feel prepared to identify or manage mental health concerns (Koller, Osterlind, Paris, & Weston, 2004), yet mental health issues among children and youth is on the rise (Fombonne, 1998). Among elementary school teachers, 91% indicated they were concerned about a student’s family stressors, 76% about anxiety, and 54% about depression; 94% also agreed that schools should be involved in addressing mental health issues. Further, teachers’ beliefs that they could help students depended on their own positive psychological wellbeing, satisfaction with school climate, and confidence (Sisask et al., 2014), highlighting further potential benefits of MHFA through promoting close relationships with community mental health and a positive school climate. The current study seeks to train all school staff to provide MHFA; the climate specialists will conduct periodic MHFA ‘boosters,’ and serve as a resource to teachers and students.

#### Environmental design

The CDC (Centers for Disease Control and Prevention (CDC), 2019) identifies environmental design as an area of interest for school violence prevention, with an ongoing study of Crime Prevention Through Environmental Design (CPTED). There are typically six components: surveillance, territoriality, image/management, access control, activity support, and target hardening (Cozens, Saville, & Hillier, 2005). *Surveillance* may be natural (e.g., teachers’ office overlooking the playground), formal (e.g., playground duty or police patrols), or mechanical (e.g., cameras). *Image/management* refers to maintaining or improving physical spaces (e.g., graffiti cleanup). *Territoriality* refers to promoting a “sense of ownership” by legitimate users, thus reducing the likelihood of “illegitimate” use. *Access control* limits the accessibility of potential targets. *Activity support* promotes “intended patterns” of use for public spaces (e.g., increasing pedestrian traffic). Finally, *target hardening* increases offender effort (e.g., by installing fences). Such elements may be differently considered as they best relate to promoting positive school climate in each school. In consultation with environmental design experts, school staff, and student input, the 3-PLT will work to implement actionable design changes to improve feelings of safety, beautification, ownership, and youth empowerment.

#### Surveillance

Cameras have become increasingly prevalent^3^ and are the second most common security measure (77% of schools), after locking/monitoring doors during school hours (93%) (Gray & Lewis, n.d.). While many school administrators believe cameras to be effective (Garcia, 2003), there is little evidence to suggest students share their confidence (Bracy, 2011; Brown, 2006). Cameras do not significantly reduce students’ self-reported victimization (Blosnich & Bossarte, 2011); rather, they may be associated with increased likelihood of physical victimization (Jeong, Kwak, Moon, & San Miguel, 2013) and fear of harm (Bachman, Randolph, & Brown, 2011). Other security measures (e.g., guards, metal detectors, locker checks) may increase fear of crime (Schreck & Miller, 2003). These highly visible efforts may increase fear by signaling the school must be unsafe (Schreck & Miller, 2003) or, “coercive” measures may contribute to an “atmosphere of mistrust (Brown, 2006).” As a whole, evidence suggests that such measures may not be particularly effective in increasing school safety; by contrast, other CPTED components such as territoriality or image/management remain more promising.

#### Image/management

Though the impact of school physical environment on safety remains relatively understudied (Johnson, 2009), evidence suggests it has merit. Wilcox et al. (Wilcox, Augustine, & Clayton, 2006) found that school disorder (e.g., presence of graffiti, litter) had a significant positive association with teachers’ perceptions of student misconduct. Notably, there was a significant negative association between teacher perceptions of school crime and hallway territoriality (i.e. teachers perceived less crime in areas with signs of ownership such as trophy cases or murals).

#### Territoriality and undefined space

CPTED strategies often address undefined space^33^ that include semi-public areas without clear ownership, “that may not be seen as anyone’s responsibility to monitor or maintain (Astor, Meyer, & Pitner, 2001).” Astor et al. (2001) used mapping and individual interviews to identify areas that elementary and middle school students perceived as unsafe. Often, these areas had characteristics of undefined space, with a lack of adult supervision and overcrowding. Astor et al. (2001) recommended additional monitoring strategies: for example, having teachers stand in their classroom doorways and greet students in the hall during transition times. Hallway supervision by staff (other than security guards) was the only security measure that effectively reduced any form of peer victimization (compared to student ID badge requirements, cameras, security guards, or a formal student code of conduct) (Blosnich & Bossarte, 2011).

#### Implementation facilitation

**Fig. 3:**
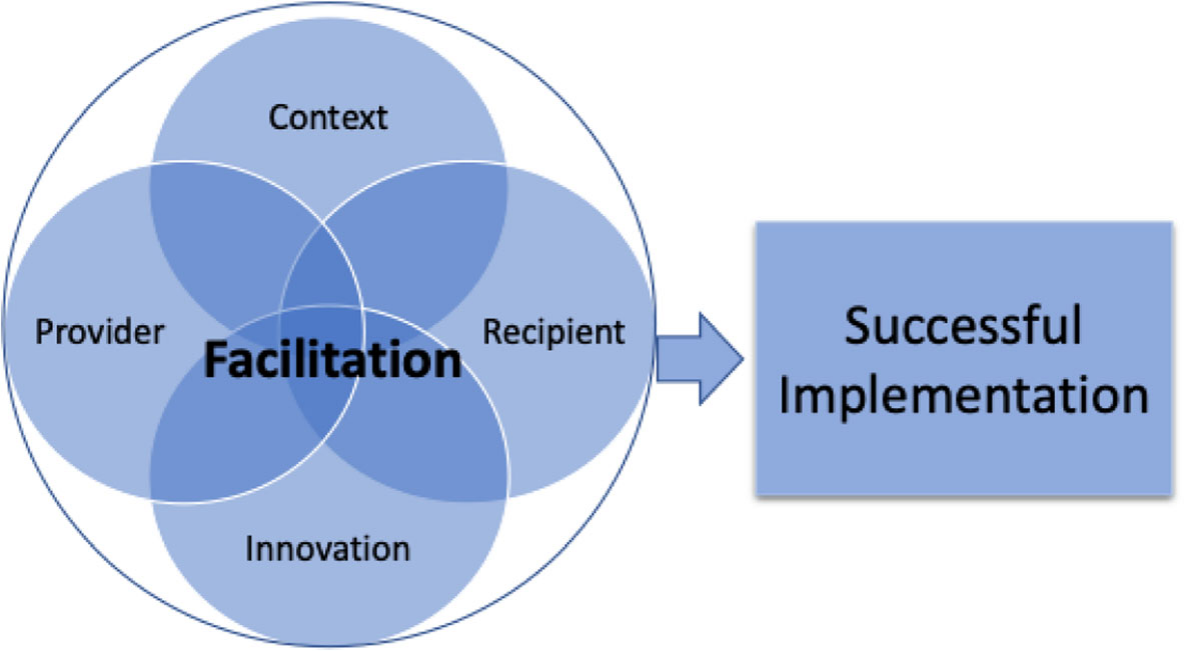
Implementation facilitation guided by the iPARHIS framework (Harvey & Kitson, 2016), adapted

Facilitation is an implementation strategy based on the Integrated-Promoting Action on Research Implementation in Health Services Framework (iPARiHS) (Harvey & Kitson, 2016; Kilbourne et al., 2013; Ritchie et al., 2014) that promotes provider self-efficacy (Bandura, 1977) in mitigating organizational barriers to EBI adoption (see Fig. 3). Facilitators are individuals who are familiar with the EBIs and organization’s procedures, climate, and processes with devoted time to support implementation activities; Facilitation includes diverse, implementation-science informed, tailored activities that enhance EBI delivery (e.g., stakeholder engagement) and identify and solve implementation challenges (Ritchie et al., 2014). Facilitation will be delivered via regular contact with the school staff and other 3-PLT members by the CS trained in program implementation and use of RJ, MHFA and CPTED in schools. The CS will support the school staff and 3-PLT in strategic thinking and program specific skills to address barriers related to the context, innovation, provider and recipients (see Fig. 3).

Although the 3-component intervention approach is consistent across schools, approaches to integrating the EBIs will be tailored, making complete standardization neither feasible nor desirable. The proposed study will include development of an implementation guide as part of the Facilitation implementation strategy. The implementation guide will provide scaffolding for the CS to work with the other 3-PLT members and school staff to integrate the interventions into each school setting. The guide will provide specific steps in delivering the EBIs across sites, but will allow for tailoring of the interventions (innovations) to meet the needs of the schools (context), their staff (providers) and their students (recipients) (Bonell et al., 2010; Patton, Bond, Butler, & Glover, 2003; Toumbourou et al., 2007); we therefore seek to evaluate district-wide (yet individualized) implementation of the three school safety components.

The CS, in collaboration with the 3-PLT will be provided with a comprehensive set of resources and training for components of MHFA, RJ, and CPTED. The 3-PLT will be trained to deliver MHFA training to all school staff, deliver restorative justice efforts (e.g., be trained in peer mediation and similar restorative processes and facilitate this within schools), and liaise with expert consultants of environmental design. Environmental design components will be tailored for each school’s unique physical and social structures; in general, aspects of the school’s image/management, and increasing ownership of undefined spaces will be emphasized. The CS will coordinate activities and efforts between the 3-PLT, school staff, parents and youth. The CS will also develop processes based on local evidence. These processes will aid in informing refinements to the implementation guide that will support program fidelity while guiding customization of adaptable elements, and long-term sustainability.

### Measures

A summary of the measures is provided in Table 2.

**Table 2:** Data sources and measures

Aim	Measures	Measure frequency	Data Sources	Original Data Sources
Primary: Intervention effectiveness on violence outcomes	Aggression, violence victimization, bullying	Fall Year 1, Spring Year 1, Fall Year 2, Spring Year 2	Michigan Profile for Healthy Youth Survey (MiPHY)^a^	Youth Risk Behavior Surveillance Survey (YRBSS)^b^ Bully-Free Schools Survey (BFSS)^c^
	Truancy, behavioral referrals	Spring Year 1, Fall Year 2, Spring Year 2	School Information System (SIS)	School Student Data
Secondary: feasibility and potential utility of implementation facilitation	Meeting agenda, interview prompts	Spring Year 1, Spring Year 2	Staff, student and 3-Person Leadership Team (3-PLT) focus groups	Questions developed by research team
	School climate, perceptions of program effectiveness (e.g., violence)	Spring Year 1, Spring Year 2	Delaware School Climate Teacher/Staff Survey^d^	Delaware School Survey - Teacher/Staff (DSS-T/S)^d^
Secondary: mechanisms and mental health	School climate scale	Fall Year 1, Spring Year 1, Fall Year 2, Spring Year 2	Delaware School Climate Student Survey^d^	Delaware School Survey - Student (DSS-S)^d^
	Anxiety, depressive symptoms, wellbeing	Fall Year 1, Spring Year 1, Fall Year 2, Spring Year 2	Michigan Profile for Healthy Youth Survey (MiPHY)^a^	Youth Risk Behavior Surveillance Survey (YRBSS)^b^
Secondary: Cost-benefit	Intervention (e.g.,materials) and imple-mentation costs (e.g., training, support)	Monthly	Time tracking by research and school staff	Data collected by research team

^a^(Michigan Department of Education, 2016), ^b^ (Kann et al., 2018), ^c^ (Lindenberger, 2005), ^d^ (Bear et al., 2016)

### Primary outcomes

#### Violence

We will use the Michigan Profile for Healthy Youth (MiPHY) to evaluate student outcomes related to aggression and violence, and the intermediate school district across Genesee Co. The MiPHY represents the Michigan Youth Risk Behavior Survey (YRBS), part of a nationwide surveying effort led by the Centers for Disease Control and Prevention (CDC) to monitor students’ health risks and behaviors (Michigan Department of Education, 2016). YRBS has been conducted annually by the CDC since 1993 and the psychometric properties have been evaluated and published (Brener et al., 2002; Kann et al., 2018). The MiPHY was designed to track students over time and those that move across districts. As a coordinated survey, MiPHY assesses a broad range of content related to students’ physical and mental health as well as attitudes, beliefs, and behaviors in their school, home, and neighborhood environments. Related to this project, it will include measurement of aggressive behavior, violence victimization, and bullying. The data will be maintained by GISD and provided in a de-identified format to the evaluation teams.

#### School-level discipline

The School Information System (SIS) is a web-based information system, implemented in GISD schools along with PBIS. It is designed to be an efficient, reliable, and confidential tool for collecting, summarizing, and using student discipline data, enabling school personnel to be more successful decision makers. Presently, schools in Genesee Co. report student violations and truancy via SIS, creating a standardized and comparable metric across schools in the proposed study area. We will compare treatment versus control school outcomes utilizing these data. Additional time will be allocated for more intensive analyses and linkage with MiPHY and focus group results.

### Secondary outcomes

#### Feasibility and utility of implementation facilitation

##### Focus groups

We will invite all teachers of senior elementary students to participate in a focus group at each intervention school at the end of the school year, for each year of the intervention. A single discussion will be held at each school at a convenient time (e.g., lunch break). We estimate 85% of teachers will participate, based on previous school research. We will ask teachers about their experiences with the intervention and the implementation supports, including their perceptions of effectiveness. We will also ask about any feedback they have on the interventions and their delivery. Discussions will be approximately 30 min in duration and will be conducted by research staff trained in facilitating focus groups. Teachers will provide informed consent prior to participation. Students from four randomly selected intervention classes will be recruited to participate each year of intervention implementation (approx. *n* = 160, 6–9 students per group). All students with parental consent/student assent who are present on the day will be invited to participate for approximately 30 min. We will ask students about their perceptions of the interventions, including satisfaction. To maintain confidentiality, no identifying details will be collected. Focus groups will be conducted at the end of each the school years by research staff trained in facilitating focus groups (including previous work with children). Finally, we will invite members of the 3-PLT to participate in focus groups and provide feedback regarding barriers and facilitators to implementation, and suggestions for how the implementation process can be improved.

##### Teacher surveys

Each spring (April–May), we will open an online survey to examine school climate from teachers’ perspectives. The survey will consist of items drawn from the teacher and staff version of the Delaware School Climate Survey (Bear, Gaskins, Blank, & Chen, 2011), which covers perceptions of safety, student behavior, and overall climate. In the original student school climate survey, researchers conducted a multigroup confirmatory factor analysis and identified multiple specific factors associated with school climate, including teacher-student relationships, school safety and liking school, using this brief, psychometrically sound scale (Bear et al., 2011). In addition, we will adapt violence and mental health items from the MiPHY to reflect teacher perceptions of the prevalence and severity of violent behaviors and mental health problems among their student populations. All teachers will be notified of the survey during a staff meeting. Reminders will be sent by the 3-PLT until a 75% response rate has been achieved.

##### Mechanisms

We will assess potential mechanisms by which participation in the intervention influences secondary student outcomes including emotional functioning (anxiety and depression symptoms, wellbeing) using items from the MiPHY survey (Michigan Department of Education, 2016). Specifically, we will examine if school climate mediates the relationship between intervention participation and school climate; school climate will be assessed using items from the Delaware survey (Bear et al., 2011). School climate serves as a critical intermediary between the intervention components and student mental health and safety experience. Collectively, the interventions are thought to improve positive perceptions of school climate across constituents – students, teachers, school staff and administrators. Students and teachers often have different perceptions of the same objective experiences. Although there is a clear link between student climate perceptions and positive student outcomes (Esposito, 1999; Haynes, Emmons, & Ben-Avie, 1997), there may be indirect influences on student outcomes through *teacher* perceptions of climate. Teachers who perceive poorer school climate report higher job-related stress and poorer teaching self-efficacy (Collie, Shapka, & Perry, 2012), which in turn can influence student outcomes. We assess climate holistically to understand how stakeholders across the school may influence ultimate student outcomes.

##### Cost estimates

We will estimate the resources required for the intervention and its implementation and estimate the net costs using a resource-based costing approach. We will track activities relevant to site replication or implementation costs, versus intervention development and research costs because only the former would be required of other sites who might adopt the intervention program (Hurley & Matthews, 2007; Neumann, Sanders, Russell, Siegel, & Ganiats, 2016). Specifically, we consider implementation costs to be those costs associated with replication that would be required by those adopting the program (Neumann et al., 2016). This would include components of the intervention that require tailoring for replication, recruitment or promotion costs to engage practitioners and participants, and training, supplies and labor costs for evaluation as indicated (Ritzwoller, Sukhanova, Gaglio, & Glasgow, 2009).

### Analysis

Our primary hypothesis is that students in schools receiving the intervention will report less violence (over time) compared to students in the control group. We also hypothesize that the intervention schools have an overall lower rate of violence over the school year compared with control schools. We expect that using implementation facilitation led by a climate specialist as part of a 3-person leadership team is a feasible and sustainable approach to integrating and effectively delivering the three intervention components. Finally, we expect that students in the intervention schools will report more positive school climate compared with those in control schools and that this, in turn, will result in lower improved mental health outcomes.

A cluster randomization design is proposed with random assignment by school to one of two arms: intervention or control. Evaluation of effectiveness outcomes will involve testing at four time points: a fall of Year 1, spring of Year 1, fall of Year 2, spring of Year 2. Measures will include school records and self-report data from students and teachers. The survey of students makes use of currently collected MiPHY data. Ten schools will be recruited in the first year and 10 in the following year; staggered recruitment will allow some initial evaluation testing of the program and allow the earliest possible start time with a number of schools. Including 20 schools in the research provides an opportunity to examine school level differences. To adjust for bias introduced by multiple comparisons across our outcome variables, we will use a pair of conservative (Tukey and Bonferonni) *p*-value adjustments in addition to reporting direct effect sizes (e.g., group differences in means) to allow for assessment not dependent on standard null hypothesis testing.

### Power analyses

#### Between group analyses

Since the proposed intervention will be delivered within group (school) settings, data analyses examining treatment effects at the school level must account for the correlation among observations within school (i.e., intraclass correlations; ICC) due to shared factors within a school setting such as school selection factors (e.g., school attendance boundaries, family choice), similarities in treatment experiences (e.g., staff factors, resource factors), and within-school influences (e.g., common experiences with other students, cultural norms). Previous analyses of measures to be used in this study indicate small ICCs within schools for our outcome variables of interest (range: .00 to .03). If an ICC greater than zero is ignored, the outcome variance due to between-school differences (which can be large) is mixed with outcome variance due to between-participants variability within schools (which can be small because of the correlation). This can lead to large standard deviations, larger *p* values, and false-negative results (Chuang, Hripcsak, & Heitjan, 2002; Murray, Phillips, Bimbaum, & Lytle, 2001). Thus, our analytic strategy described below takes the ICC into account. Assuming 20 schools, 60 students per school, and a modest within-cluster correlation coefficient (ρ = .10), we will be able to detect standardized effect sizes at the school level of *d* = .27, representing a medium effect size, with probability (β > .80). Thus, our design will include 20 schools over the course of 2 years of the study period. The lagged implementation, however, limits across school comparisons (i.e., treatment vs control schools) until year three.

#### Individual analyses

At the individual level, we will examine the hypothesized pathways using structural equation models while controlling for dependency due to school attended as noted above. Across Genesee County, there are an average of 180 4th–6th Graders in Flint schools resulting in a maximum sample size of 3600 students over the course of the study (we use a 33% consent/retention rate for calculations). Using the SEM power calculations suggested by MacCallum, Browne, and Sugawara (1996), we will have ample statistical power in SEM analyses for even our most complex models with 100 degrees of freedom (*df*). Power is based on the probability of rejecting the hypothesis of not a close fit with the data when the true model fit is excellent (the most stringent test). Our power with the 1200 (600 per condition) students is over .80. Notably, MacCallum et al. (1996)^.^ show that power of .80 is achievable with alpha = .05 and *df* of 100 with a sample as small as 178 for test of not-close fit.

#### Behavioral outcome analysis

We will undertake comparability of the two arms (intervention and control) will be undertaken, including school size and socio-demographic indicators (e.g., proportion of free/reduced school lunches, proportion of students from various ethnic backgrounds, aggregate reports of income). Our primary analytic strategy uses mixed-effects models with full information maximum likelihood, which uses all available data for point estimation and sandwich estimator for the standard errors.

##### Data linking

Survey data will be linked across intervention Years 1 and 2 for each Cohort. In order to maintain anonymous management of data, GISD will generate individual codes in the de-identified data. The impact of each year of the intervention will be assessed as well as change after 2 years of implementation. Rarely is the stability of an intervention examined, particularly one that is designed to affect change in school climate. The two-year implementation of the intervention provides a unique opportunity to assess the intervention over a longer period.

##### Dichotomous outcome variates

The outcome measure required is the net average change, i.e. the proportion changing from negative at baseline to positive at follow-up minus the proportion changing from positive at baseline to negative at follow-up. This can be modeled in multinomial form with categories—upward change (from negative for the behavior to positive for the behavior), downward change (the reverse), with no change as reference category—and analyzed using multiple logistic regression for a cluster design by e.g., the GENMOD procedure in SAS.

As a test of the conceptual framework (secondary aim), mediating variables would be included in these analyses (e.g., of school climate on mental health). Change in certain behavioral variables, including composite variables, will be correlated by a multivariate extension of the above techniques to examine the relationship between school climate, mental health, and violence.

##### Quantitative variates (e.g., violence; climate; mental health)

Essentially the same analytic structure as described above for dichotomous variates will be employed, but without the logit link for linear variates (it may be necessary to transform these quantitative variates, depending on their distributions.)

#### Implementation outcomes

We will assess feasibility and potential utility of using implementation facilitation through focus groups. A minimum of two study team members will take detailed field notes during focus group sessions. We will use the field notes to develop a broad understanding of content as it relates to the project’s specific aims and to identify topics of discussion and observation. During this and subsequent steps, we will document initial impressions of topics and themes and their relationships to each other to define the boundaries of specific codes (e.g., the inclusion or exclusion criteria for assigning a specific code; (Miles, Huberman, & Saldaña, 2014) The empirical material from the focus groups will be coded by project team members to condense the data into analyzable units. Segments of text will be assigned codes based on a priori or emergent themes, also known as open coding (Strauss & Corbin, 1998). Codes will also be assigned to describe connections between categories (e.g., barriers and feasibility). Lists of codes developed by each investigator will be matched and integrated into a single codebook. We will use these codes to examine the association between different a priori and emergent categories. Through the process of continually comparing these categories, the different categories will be further condensed into broad themes. We will also evaluate outcomes using descriptive analyses from teacher surveys and a mixed methods approach to develop a comprehensive understand of using Facilitation to deliver the interventions. Results from each data set will be examined side-by-side to explore convergence (i.e., comparing analysis conclusions) to investigate if qualitative and quantitative results concur. We will also investigate how focus group results elaborate on quantitative results (expansion) to deepen our understanding of why and how Facilitation may or feasible for comprehensive school safety in low resource communities (Palinkas et al., 2011).

#### Process evaluation

An intervention of this nature and size requires a comprehensive process evaluation to monitor implementation and sustainability. Specifically, we will focus on barriers and facilitators to implementation. We will investigate barriers and facilitators associated with the context (e.g., implementation climate), innovation (e.g., complexity), providers (e.g., confidence, competence) and recipients (i.e., student needs). We will examine key factors through focus groups with school staff, observations and review of 3-PLT meetings and activities.

#### Cost-benefit analysis

Following findings of reduced violence, we will examine the costs and benefits of the intervention. The program will be compared with the alternative intervention of no program (control). Although the intervention is targeted to reducing violence, it will likely have additional effects; this adds complexity to the analysis. We will focus on benefits associated with reduced violence, based on previously used methodology to obtain costs (Foster & Jones, 2006; Kuklinski, Briney, Hawkins, & Catalano, 2012). This strategy considers the proportion of school violence from national costs estimated for youth violence and related early school separation. School absence costs will also be considered, relative to costs associated with processing school truancy and absences associated with fear of attending school. We will compare these benefits with costs associated with implementing the full program.

### Planned dissemination

We have existing mechanisms for dissemination of study results to both research and practice communities. This includes a Center that is part of the CDC-funded network of National Centers of Excellence in Youth Violence Prevention, which may serve as an additional venue for disseminating results to researchers and practice professionals. Study team members also house a Bureau of Justice Assistance training and technical assistance programs for Project Safe Neighborhoods, the Violence Reduction Assessment Tool, and the Innovations Suite Research Practitioner Fellows Academy that provide outstanding mechanisms for the dissemination of findings to teams of practitioners and researchers across the US. Finally, we will disseminate results and lessons learned to practitioners (including school administrators and resource officers) at conferences such as Michigan’s SEPLA conference (http://www.seplainstitute.org/index.htm) and the conferences of the Michigan Chiefs of Police and Sheriffs Associations.

In addition, we will make information about this research available to a broader audience including the GISD community (students, families, staff, and local residents); local, state, and national policy makers; and the general public. Upon study completion, we will feed back results to the GISD community via newsletters, town halls, and presentations to school administrators, district officials, and the Genesee Intermediate Board of Education. We will make this information available for a common NIJ-sponsored school safety website.

## Discussion

This study will make important contributions in the areas of implementation science, prevention research, violence prevention and the health and well-being of youth in disadvantaged communities. Although implementation science has made notable gains in the translation of EBIs many areas of public health and clinical science, including mental health treatment and cancer prevention, implementation is understudied in violence prevention and school-based EBIs. In addition, this study makes an important contribution to the application of implementation strategies to reduce health disparities through the effective delivery of EBIs in disadvantaged communities. This study is one of the first of its kind to investigate the feasibility of applying an implementation facilitation approach to deliver a comprehensive, multicomponent violence prevention intervention in schools.

The current study will evaluate the effectiveness of a comprehensive intervention to improve three key areas that affect school safety: violence, student mental health, and climate. Results from both the outcomes and process evaluations are expected to have significant implications for criminal justice policy and practice. In particular, this study emphasizes a proactive approach with early intervention, intended to prevent students from negative outcomes such as delinquency, violence, or psychiatric disorders later in life. It also emphasizes a community-based approach (including links with local health services) and a proactive and positive supportive approach (e.g., team-based supervision, restorative justice) over exclusionary discipline, in order to avoid a school to prison pipeline. This study will add to the literature by assessing both Mental Health First Aid and Restorative Justice among relatively young students, including new information about barriers to implementation for approaches that are not well studied in terms of their fidelity. The proposed study will also address less commonly considered components of CPTED, which may provide schools with a lower cost alternative to security cameras. It expands the role of school safety officers, who will serve not just as a visible police presence at school, but as key members of a leadership team that is integrated into the school community. This leadership team in particular is intended as a model for sustainable interventions that focus on building school capacity to improve safety. Finally, the study sample involves high need youths and schools reflective of disadvantaged communities subject to significant safety concerns and at-risk for the school to prison pipeline, absent interventions such as those proposed. This study aims to create a comprehensive, well-integrated model intervention that may be implemented in similar high-risk settings.

The current study will also add to our understanding of implementing multicomponent interventions for school safety in terms of logistical, social and material resource challenges. When delivering multiple interventions simultaneously, some may be more (or less) straightforward to integrate, and/or feasible and acceptable. This is especially critical for schools located in communities serving youth at high risk of poor health outcomes with significant constraints on time and resources. Consequently, this research can provide valuable information about options for schools to integrate these interventions (e.g., simultaneously, staggered), and secure needed social and material resources (e.g., substitute teachers for trainings) for successful implementation. This project will also advance community-research partnerships for school safety and in implementation science. The selection of interventions and implementation strategy represent a collaboration between the research partners, the intermediate school districts and the individual schools. Finally, this project seeks to support sustainability through involving school and district partners in each step of the process, adding personnel in the schools to coordinate implementation efforts and building capacity of school staff to conduct training and support intervention delivery over the long-term.

## Data Availability

De-identified quantitative survey data, notes from qualitative interviews and transcripts from focus-group data will be provided to the National Archive of Criminal Justice Data (NACJD) at ICPSR at the University of Michigan within 2 years after the completion of the project. The NACJD will make the final determination of whether or not the data are suitable for public release, as well as the conditions associated with the release of the data if it is deemed appropriate for public use. Requests for the data can be made through their online data portal found at: https://www.icpsr.umich.edu/icpsrweb/content/NACJD/index.html.

## Abbreviations

3-PLT: 3-Person Leadership Team
CDC: Center for Disease Control and Prevention
CPTED: Crime Prevention Through Environmental Design
CS: Climate Specialist
EBI: Evidence-Based Interventions
Enhanced REP: Enhanced Replicating Effective Programs
GENMOD: Statistical procedure used for solving generalized linear equations
GISD: Genesee County Intermediate School District
iPARiHS: Integrated-Promoting Action on Research Implementation in Health Services
MHFA: Mental Health First Aid
MIBLISI: Michigan’s Integrated Behavior and Learning Support Initiative
MiPHY: Michigan Profile for Health Youth
NIJ: National Institute of Justice
PBIS: Positive Behavioral Interventions & Support
RJ: Restorative Justice
SAMSHA: Substance Abuse and Mental Health Services Administration
SAS: Statistical Analysis System
SEPLA: Schools, Educators, Police, Liaison Association
SIS: School Information System
SRO: School Resource Officer
